# PlaPPISite: a comprehensive resource for plant protein-protein interaction sites

**DOI:** 10.1186/s12870-020-2254-4

**Published:** 2020-02-06

**Authors:** Xiaodi Yang, Shiping Yang, Huan Qi, Tianpeng Wang, Hong Li, Ziding Zhang

**Affiliations:** 1 State Key Laboratory of Agrobiotechnology, College of Biological Sciences, China Agricultural University, Beijing, 100193 China; 2 Key Laboratory of Tropical Biological Resources of Ministry of Education, School of Life and Pharmaceutical Sciences, Hainan University, Haikou, 570228 China

**Keywords:** Plant, Database, 3D structures of protein complexes, Protein-protein interaction site, Domain-domain interaction, Domain-motif interaction, Interolog

## Abstract

**Background:**

Protein-protein interactions (PPIs) play very important roles in diverse biological processes. Experimentally validated or predicted PPI data have become increasingly available in diverse plant species. To further explore the biological functions of PPIs, understanding the interaction details of plant PPIs (e.g., the 3D structural contexts of interaction sites) is necessary. By integrating bioinformatics algorithms, interaction details can be annotated at different levels and then compiled into user-friendly databases. In our previous study, we developed AraPPISite, which aimed to provide interaction site information for PPIs in the model plant *Arabidopsis thaliana*. Considering that the application of AraPPISite is limited to one species, it is very natural that AraPPISite should be evolved into a new database that can provide interaction details of PPIs in multiple plants.

**Description:**

PlaPPISite (http://zzdlab.com/plappisite/index.php) is a comprehensive, high-coverage and interaction details-oriented database for 13 plant interactomes. In addition to collecting 121 experimentally verified structures of protein complexes, the complex structures of experimental/predicted PPIs in the 13 plants were also constructed, and the corresponding interaction sites were annotated. For the PPIs whose 3D structures could not be modelled, the associated domain-domain interactions (DDIs) and domain-motif interactions (DMIs) were inferred. To facilitate the reliability assessment of predicted PPIs, the source species of interolog templates, GO annotations, subcellular localizations and gene expression similarities are also provided. JavaScript packages were employed to visualize structures of protein complexes, protein interaction sites and protein interaction networks. We also developed an online tool for homology modelling and protein interaction site annotation of protein complexes. All data contained in PlaPPISite are also freely available on the Download page.

**Conclusion:**

PlaPPISite provides the plant research community with an easy-to-use and comprehensive data resource for the search and analysis of protein interaction details from the 13 important plant species.

## Background

Proteins are involved in most biological processes in cells, and they tend to perform their biological functions in stable or transient complexes rather than in isolation [1]. Therefore, the large-scale identification of protein-protein interactions (PPIs) is an important step to globally understand the landscape of the whole proteome. To date, a large number of high-throughput experiments have been employed to identify genome-wide PPIs (also termed interactomes) in model organisms such as *Arabidopsis thaliana, Saccharomyces cerevisiae, Caenorhabditis elegans, Drosophila melanogaster, Homo sapiens* and *Escherichia coli K12* [2–7]*.* Regarding the known PPI inventory in plants, 36,099 *A. thaliana* PPIs have been deposited in BioGRID (v3.4.155) [8]. By contrast, the number of known PPIs from other plants is limited since experimental methods are still time-consuming and laborious.

To improve the coverage of PPIs, a variety of computational methods have been developed to predict PPIs, including interolog mapping [9, 10], gene/domain fusion-based PPI inference [11, 12], domain-domain/motif interaction transfer [13, 14], gene co-expression [15], machine learning approaches [16, 17], etc. These methods have also been widely applied to predict plant PPIs [18, 19], and some helpful data resources have been available for plant scientists to further investigate the functional mechanisms of plant proteins [20–24].

To further decipher the molecular mechanisms of PPIs, a key step is to identify interaction domains, motifs and sites associated with PPIs. Some databases have integrated the information of interaction domains and motifs from Protein Data Bank (PDB) [25], for example, the database of 3D interacting domains (3did) [26]. Protein interaction sites can be identified from experimentally verified structures of protein complexes. However, only approximately 120 non-redundant heterodimers for plants were available in the 2018 release of PDB, which lags far behind the number of experimentally verified plant PPIs [8, 27–30]. Therefore, bioinformatics methods will play an important role in accelerating the annotation of interaction domains, motifs and sites for both experimental and predicted PPIs.

In 2016, we developed AraPPISite [31] to provide detailed information about 7336 experimentally determined PPIs for the model plant *A. thaliana*. AraPPISite allows researchers to query the 3D structures, protein interaction sites, DDIs and DMIs of PPIs. Moreover, it displays abundant physicochemical annotations of interaction sites. However, AraPPISite has certain limitations. First, AraPPISite only takes one organism, *A. thaliana*, into account. Second, AraPPISite only provides protein interaction details of limited experimentally verified PPIs while ignoring the predicted PPIs, which narrows the coverage of AraPPISite. Moreover, the number of experimental PPIs has greatly increased after the publication of AraPPISite. Third, AraPPISite lacks a convenient prediction platform for protein complex structure construction and interaction site assignment, which is not convenient when the query PPIs are not present in AraPPISite. In this context, it is necessary to evolve AraPPISite into a new version that provides interaction details with higher coverage for multiple plant interactomes.

Here, we present PlaPPISite (http://zzdlab.com/plappisite/index.php), a free and user-friendly database of plant protein interaction sites. Compared to its precedent version (i.e., AraPPISite), PlaPPISite incorporates 12 other plant interactomes. Although the PPI networks and the corresponding interaction sites are mainly inferred from computational methods, PlaPPISite greatly increases the coverage of PPIs with interaction site annotations. Moreover, a convenient prediction platform was integrated into PlaPPISite, in which users could merely submit a pair of protein sequences to obtain the protein complex structure and interaction site information.

## Construction and content

### Database architecture

**Fig. 1:**
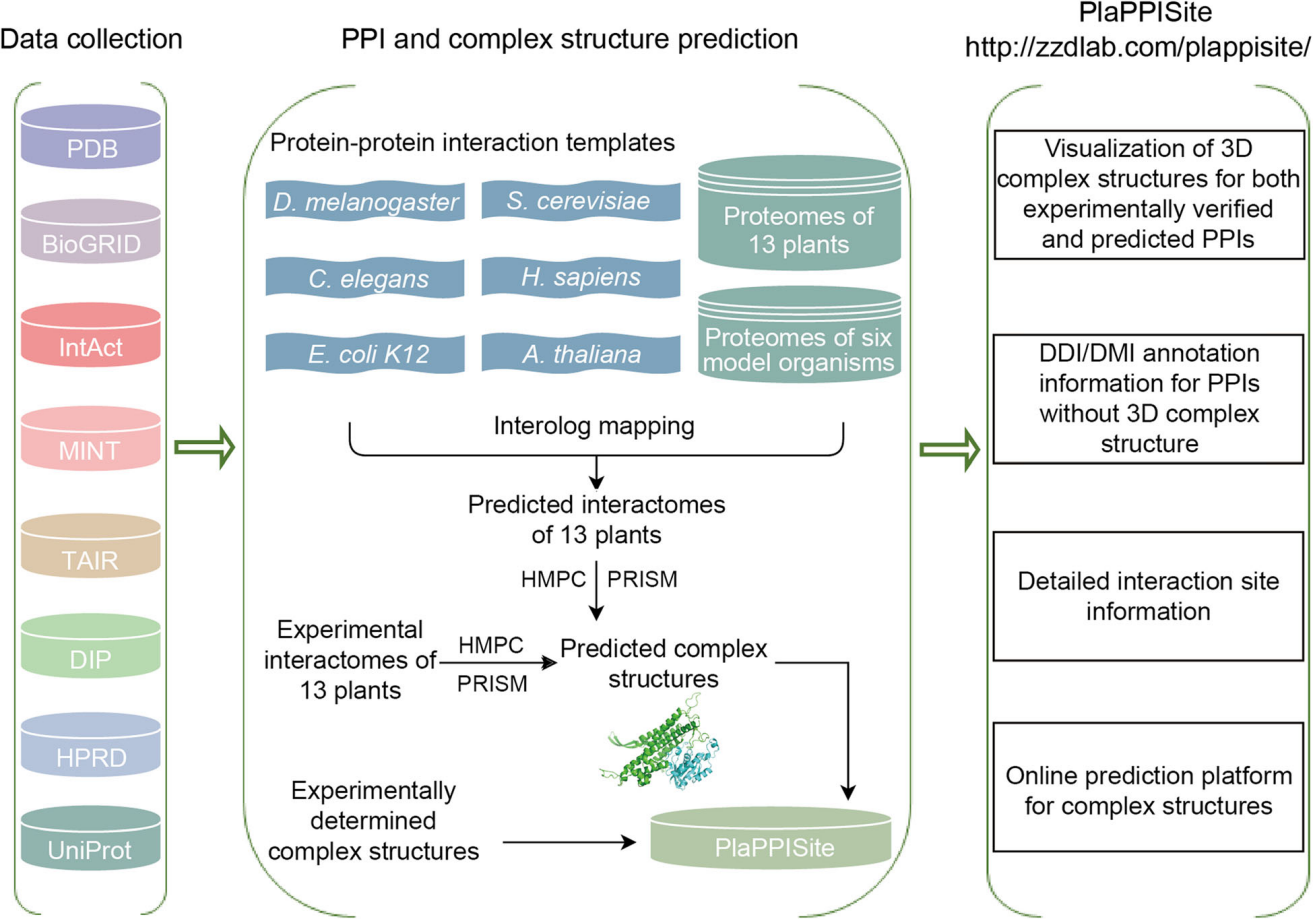
The flowchart of database construction

**Fig. 2:**
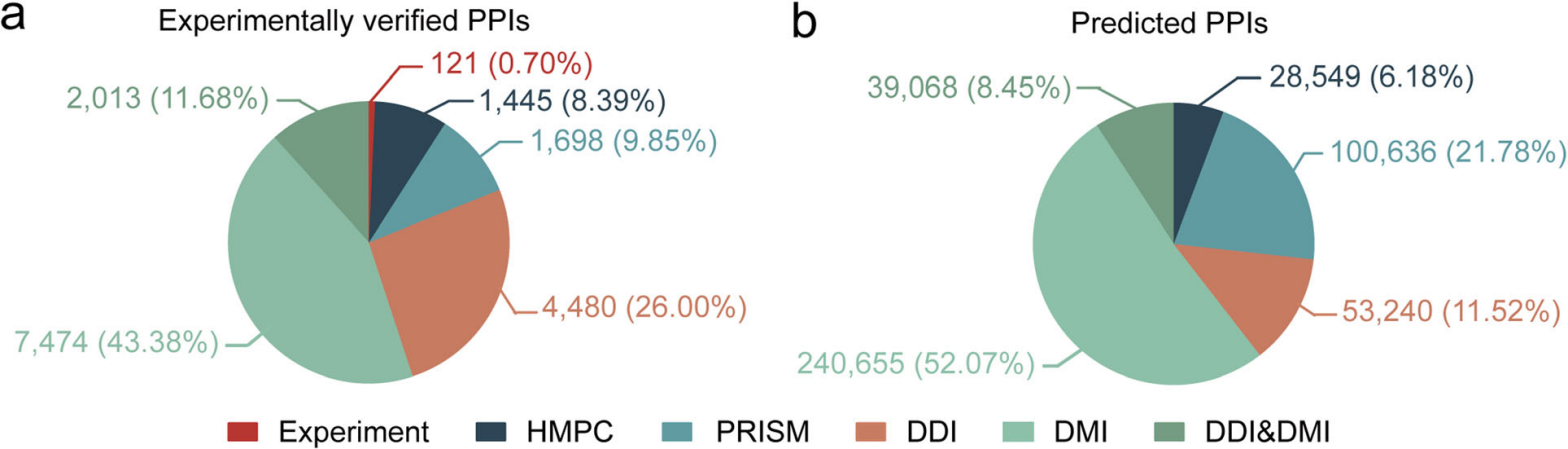
The proportions of different annotation information in experimentally verified (**a**) and predicted (**b**) PPIs

The flow chart for constructing PlaPPISite is described in Fig. 1. The current PlaPPISite contains 17,231 experimentally verified PPIs and 462,148 predicted PPIs. The distribution of PPIs in PlaPPISite is shown in Fig. 2 and Additional file 1: Table S1. Among the 17,231 experimentally verified PPIs, only 121 have experimentally verified structures of protein complexes, which were deposited in the PDB database. By using Homology Modelling of Protein Complex (HMPC) and Protein Interactions by Structural Matching (PRISM), we obtained the predicted structures of protein complexes of 1445 and 1698 PPIs, respectively. The remaining 13,967 experimentally verified PPIs were only annotated with DDIs/DMIs. For the 462,148 predicted PPIs, HMPC and PRISM successfully predicted 28,549 and 100,636 structures of protein complexes, respectively. The remaining 332,963 PPIs were also annotated with DDIs/DMIs.

### Collection and processing of experimentally verified PPIs

We collected the experimentally verified PPIs of 13 plants, including *A. thaliana*, *Chlamydomonas reinhardtii*, *Ricinus communis*, *Glycine max*, *Oryza sativa*, *Selaginella moellendorffii*, *Solanum lycopersicum*, *Solanum tuberosum*, *Vitis vinifera*, *Zea mays*, *Brachypodium distachyon*, *Populus trichocarpa* and *Medicago truncatula* from five public databases (BioGRID, https://thebiogrid.org/ [8]; IntAct, https://www.ebi.ac.uk/intact/ [28]; MINT, https://mint.bio.uniroma2.it/ [27]; DIP, https://dip.doe-mbi.ucla.edu/dip/Main.cgi [30]; TAIR, https://www.arabidopsis.org/ [29]). The self-interactions, redundant interactions and non-physical interactions were deleted. To unify protein IDs for these 13 plants, different types of protein IDs were converted to UniProt IDs. As a result, 49,007 non-redundant PPIs of the 13 plants were obtained (Additional file 1: Table S2).

### Genome-wide prediction of plant PPIs

Compared with *A. thaliana*, which contains 48,607 experimentally verified PPIs, the experimentally verified PPIs of the other 12 plants are rare. To complement the experimentally verified PPIs, genome-wide PPI predictions of these 13 plants were carried out through interolog mapping method [9]. Briefly, two proteins (*A* and *B*) in one of the plants can be predicted to interact with each other in case an experimentally validated PPI exists between their respective orthologous proteins (*A’* and *B′*) in other species. The protein pair (*A’*, *B′*) is also regarded as the interolog template of the protein pair (*A*, *B*). To obtain high-quality interolog templates for the prediction of plant PPIs, we first collected experimentally verified PPIs of six model organisms, including *A. thaliana*, *S. cerevisiae*, *C. elegans*, *D. melanogaster*, *H. sapiens* and *E. coli K12*, from BioGRID, IntAct, MINT, DIP, TAIR and HPRD [32] (Additional file 1: Table S3). Then, the protein sequences of the model organisms and the 13 plants were downloaded from the UniProt database [33]. Moreover, InParanoid 8 [34] was used to identify the orthologs between the 13 plants and the model organisms. To ensure the quality of predicted PPIs, a stringent threshold (i.e., the InParanoid score = 1.0) used in [35] was employed to infer the orthologous relationship. As a result, the predicted protein interactomes of these 13 plants were generated through interolog mapping, and the corresponding number of PPIs for each plant is shown in Table 1.

**Table 1:** The number of predicted PPIs in the 13 plants of PlaPPISite

Organism	The number of predicted PPIs
*A. thaliana*	104,009
*C. reinhardtii*	49,350
*R. communis*	99,157
*G. max*	160,024
*O. sativa*	99,296
*S. lycopersicum*	110,943
*S. tuberosum*	81,057
*V. vinifera*	105,415
*Z. mays*	112,597
*S. moellendorffii*	112,480
*B. distachyon*	105,705
*P. trichocarpa*	135,876
*M. truncatula*	112,478
Total	1,388,387

### Reliability assessment of predicted protein interaction networks

**Fig. 3:**
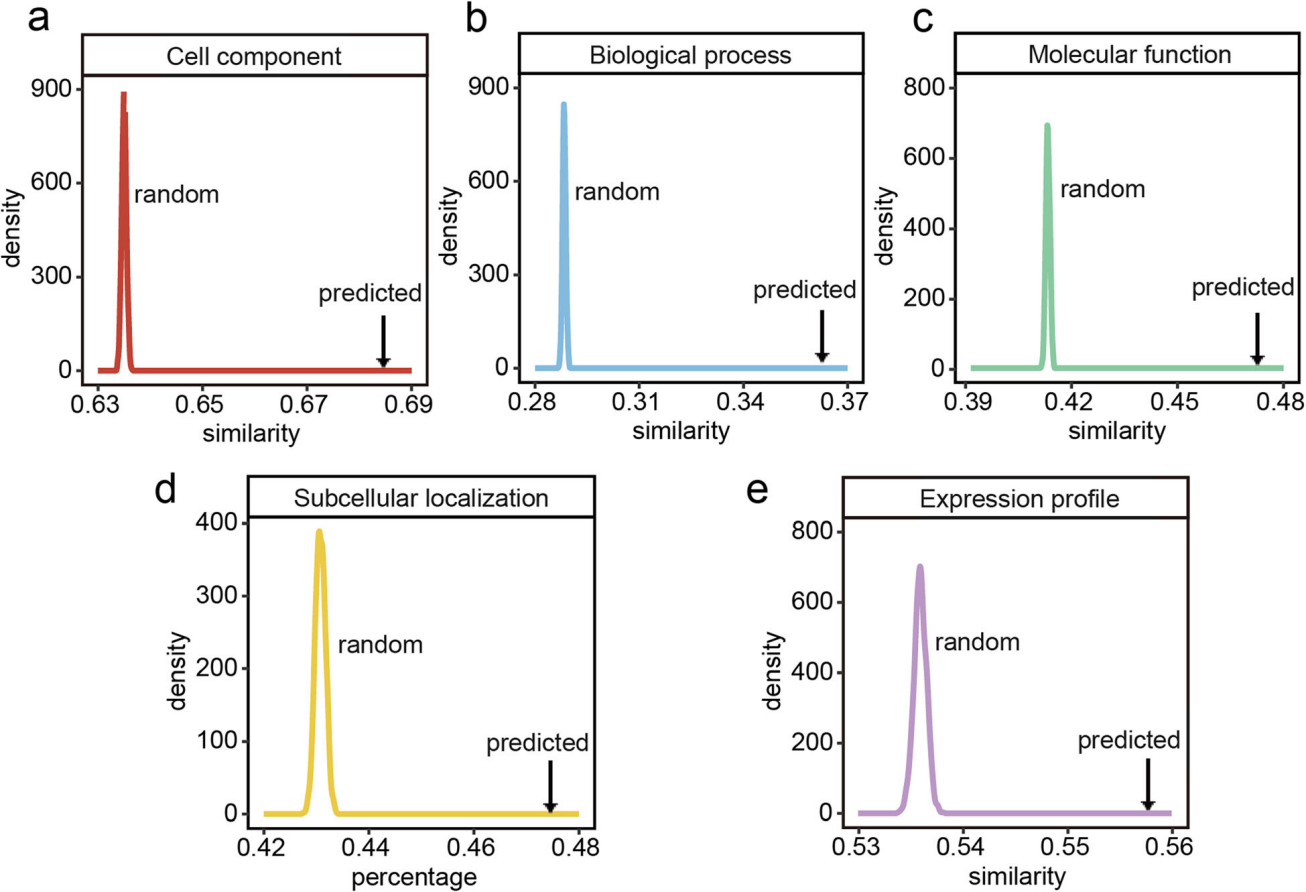
The reliability assessment evidence for the predicted *A. thaliana* PPIs*.* **a**-**c** The distribution of the average GO term similarities for 1000 random networks and the predicted PPI network. **d** The distribution of the average subcellular co-localization proportions for 1000 random networks and the predicted network. **e** The distribution of the average gene expression similarities for 1000 random networks and the predicted network

Due to the general lack of sufficient experimentally verified plant PPIs, indirect evidence including the similarities of Gene Ontology (GO) terms, the proportions of subcellular co-localization and the similarities of gene expression profiles were used to assess the reliability of the 13 predicted protein interactomes. As an important gene functional annotation system, GO annotation consists of three categories, i.e., molecular function, cellular component and biological process. It has been reported that two proteins sharing similar GO annotations have higher possibility to interact with each other. We downloaded GO annotations of these 13 plants from the GO database [36, 37] and mapped them to the 13 predicted interactomes. The GO annotations in the GO database were inferred from a variety of evidence, including experimental and computational evidence. Indeed, some GO terms were annotated through orthologous relationships. For each predicted plant interactome, high-coverage GO annotations were obtained (Additional file 1: Table S4). Moreover, an R package called GOSemSim [38] was applied to calculate the GO similarity between any two interacting proteins. To evaluate the reliability of the predicted PPI networks, 1000 random networks were constructed for each plant based on the corresponding predicted interactome by using an R package called igraph [39]. The function keeping_degseq was employed to randomly rewire the edges while preserving the original degree distribution of the network. Moreover, the similarities of GO terms were re-calculated for each random network. As a result, the average GO similarity in the predicted network is significantly higher than those in 1000 random networks constructed for each plant, meaning that the 13 predicted networks are of acceptable reliability. For instance, the average GO similarity of the predicted *A. thaliana* network is higher than that of any random network (empirical *P*-value < 0.001; Fig. 3a-c). The same trend was observed in the other 12 plants (Additional file 2: Figures S1-S3).

It has been established that interacting proteins tend to have the same subcellular localization (i.e., co-localization). Considering that most plants lack proteome-wide subcellular localization information, we predicted the subcellular localizations of proteins for 13 plants through a popular predictive tool called MultiLoc2 [40], which provides a specialized prediction module for plant proteins. The predictions of MultiLoc2 cover 10 subcellular localizations, including nuclear, cytoplasm, mitochondria, chloroplast, extracellular matrix, plasma membrane, peroxisome, endoplasmic reticulum, Golgi apparatus and vacuole. As a result, approximately 50% of the PPIs are co-localized in each plant (Additional file 1: Table S5), which is higher than the corresponding proportion in any random PPI network (Fig. 3d, Additional file 2: Figure S4).

Moreover, transcriptome data were also applied to perform the reliability assessment [15]. Protein-coding genes that exhibit similar expression patterns across different stages or time points are more likely to interact [41]. The most commonly used co-expression measure is the Pearson correlation coefficient (PCC). In our study, we retrieved gene expression data of nine plants (*G. max*, *O. sativa*, *Z. mays*, *A. thaliana*, *S. lycopersicum*, *V. vinifera*, *B. distachyon*, *P. trichocarpa* and *M. truncatula*) from the Gene Expression Omnibus (GEO) database [42], due to lacking available expression data for the other four plants. For each plant, 20 expression profile samples from different tissues, organs or developmental phases belonging to the same genotype were manually filtered. The PCC value was calculated between any two interacting proteins based on their gene expression profiles. Likewise, the average PCC value in the predicted network was significantly higher than those in 1000 random networks constructed for the nine plants, further suggesting that the predicted PPI networks are of reasonable reliability (Fig. 3e, Additional file 2: Figure S5).

Regarding the interactome of *A. thaliana*, the number of experimental PPIs is relatively large, and some predicted interactomes have been publicly available, which has allowed us to directly compare our predicted PPIs with some existing interactomes. To this end, we collected 9065 predicted highly reliable *A. thaliana* PPIs (S-PPIs) from [43], which was based on a docking scoring algorithm using both experimentally determined and predicted protein structures. The self-interactions and interactions with proteins not appearing in our collected *A. thaliana* proteome were removed, and 8358 PPIs were finally retained. To ensure a fair comparison, we selected our predicted high-quality *A. thaliana* PPIs, which included 38,610 interolog-inferred PPIs whose 3D structures could be built up or DDIs/DMIs could be annotated. In general, the numbers of overlapping PPIs among our predicted PPIs, S-PPIs and experimental PPIs are low, but they are significantly overlapped (Additional file 2: Figure S6; hypergeometric test, all pair-wise *P*-values < 2.2 × 10^− 16^). Comparatively, our predicted PPIs share a higher overlapping rate with experimental PPIs in comparison to S-PPIs. Collectively, the above direct comparison further suggests that our predictions have a comparable and reasonable accuracy.

### Annotations of experimental/predicted complex structures and interaction sites

A total of 101 experimentally verified complex structures related to *A. thaliana*, *C. reinhardtii*, *O. sativa* and *Z. mays* were collected from the PDB database, involving 121 non-redundant binary PPIs. Considering that some PPIs may own multiple sets of complex structures from different structure determination sources, the corresponding complex structures with the best resolution were retained. If two interacting proteins could map to multiple chains of the candidate complex structure, the two chains with the largest interaction interface were further selected as the final complex structure of the PPI.

Experimental complex structures are not available for most plant PPIs. Following our previous strategy in AraPPISite, two template-based methods (HMPC and PRISM) were further applied to predict the complex structures of both experimentally verified and predicted PPIs, which mainly included three steps, i.e., template selection, monomer modelling and complex modelling.

To model the complex structure of an interacting protein pair, we first selected the best homologous template for each protein through BLAST searching [44] against the PDB database. The template candidates inferred from BLAST should have at least 30% sequence identity with the query protein, and the alignment should cover at least 40% of the sequence length of the query protein. In general, the template candidate with the highest sequence identity was considered as the best template of the query protein. In some cases, template candidates shared similar sequence identity but different alignment coverage, the one with highest alignment coverage was prioritized [45]. The other template selection criteria were the same as those used in AraPPISite. The next step was to construct a monomer model for each protein of the interacting protein pair based on the selected templates. Five models for each protein were generated by Modeller (version 9.19) [46], from which the model with the lowest DOPE score was chosen. Unaligned residues at the N- and C-termini of the protein, i.e., the residues outside the boundaries of the alignment, were truncated to ensure the quality of the predicted protein structure. Once the predicted structures of two interacting proteins were obtained, the corresponding protein complex structure was further modelled. We first used HMPC to infer the complex structure, which requires the two templates of the interacting protein pair are from two different contacting chains of the same complex structure in the PDB database. Otherwise, the PRISM software [47] was employed to infer the complex structure, which only requires the two monomer structures share a similar binding interface with known complex structures. Additional details regarding the implementations of HMPC and PRISM are available in [31].

Moreover, the interaction sites can be retrieved from the experimental/predicted complex structures. Briefly, the residues from two interacting proteins were assigned as interacting sites (residues) if their shortest atomic distance was less than or equal to 4.0 Å. All the interacting residues between two interacting proteins constitute a complete interaction interface.

### Web implementation

The database construction was based on MySQL 5.5.60 and PHP 5.4.16. The service runs on an Apache 2.4.6 server with the Linux operating system CentOS 7.4. Similar to [48], a JavaScript graph library called Cytoscape.js [49] was applied to display the PPI networks. The tables and interactive charts were generated based on several web-based JavaScript libraries, such as DataTables.js, echarts.js and Highcharts.js. NGL [50], a WebGL-based 3D viewer powered by MMTF, was used to display the 3D complex structures of PPIs.

## Utility and discussion

Our goal is to develop a comprehensive database of plant protein interaction sites that consists of multiple functional modules. It allows users to explore the associations between proteins from a systematic perspective and visualize protein complex structures and interaction sites. In the meantime, it also provides an online prediction platform. Moreover, it allows users not only to access data directly from the online database but also to download the complete data for local use.

### Data access

**Fig. 4:**
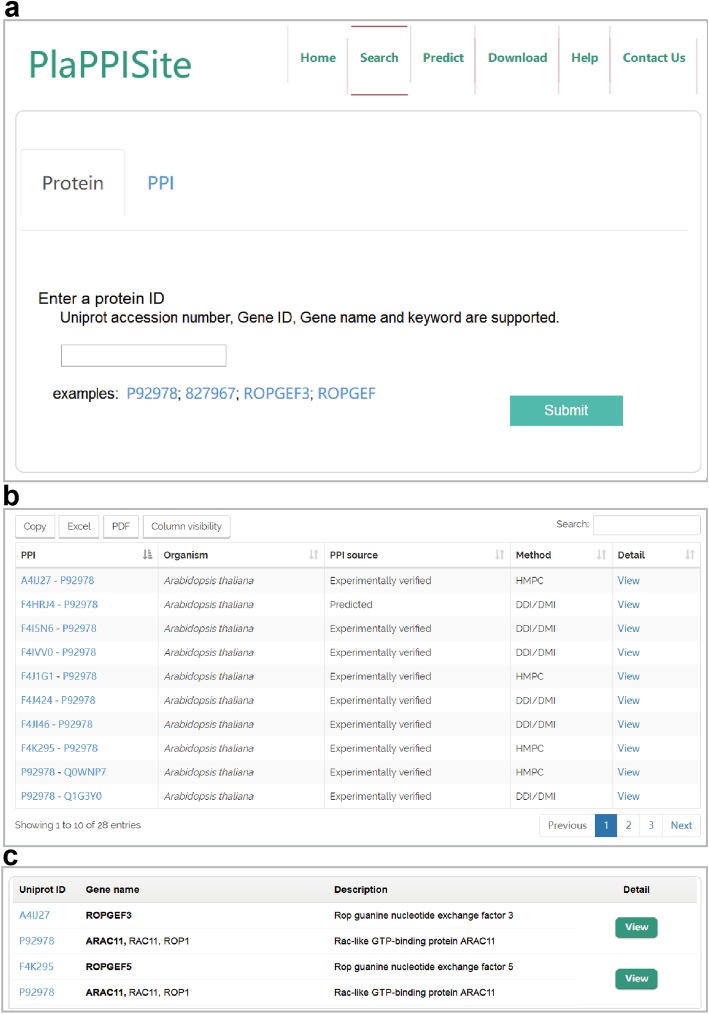
Two different ways to obtain detailed PPI information. **a** The search page in PlaPPISite. Users can not only query a single protein by inputting a UniProt ID or a keyword but also query a specific PPI directly. **b** Retrieved result for a single protein search. **c** Retrieved result for a specific PPI search

PlaPPISite provides two ways to obtain the annotation information of PPIs (Fig. 4a). Users can input a single protein ID or keyword. The PPIs associated with the query protein, source organism, PPI determination methods and the prediction method of the complex structure will be listed in a table (Fig. 4b). Likewise, users can also directly access a PPI of interest by searching two protein IDs or keywords (Fig. 4c).

### Visualization of protein complex structures and interaction details

**Fig. 5:**
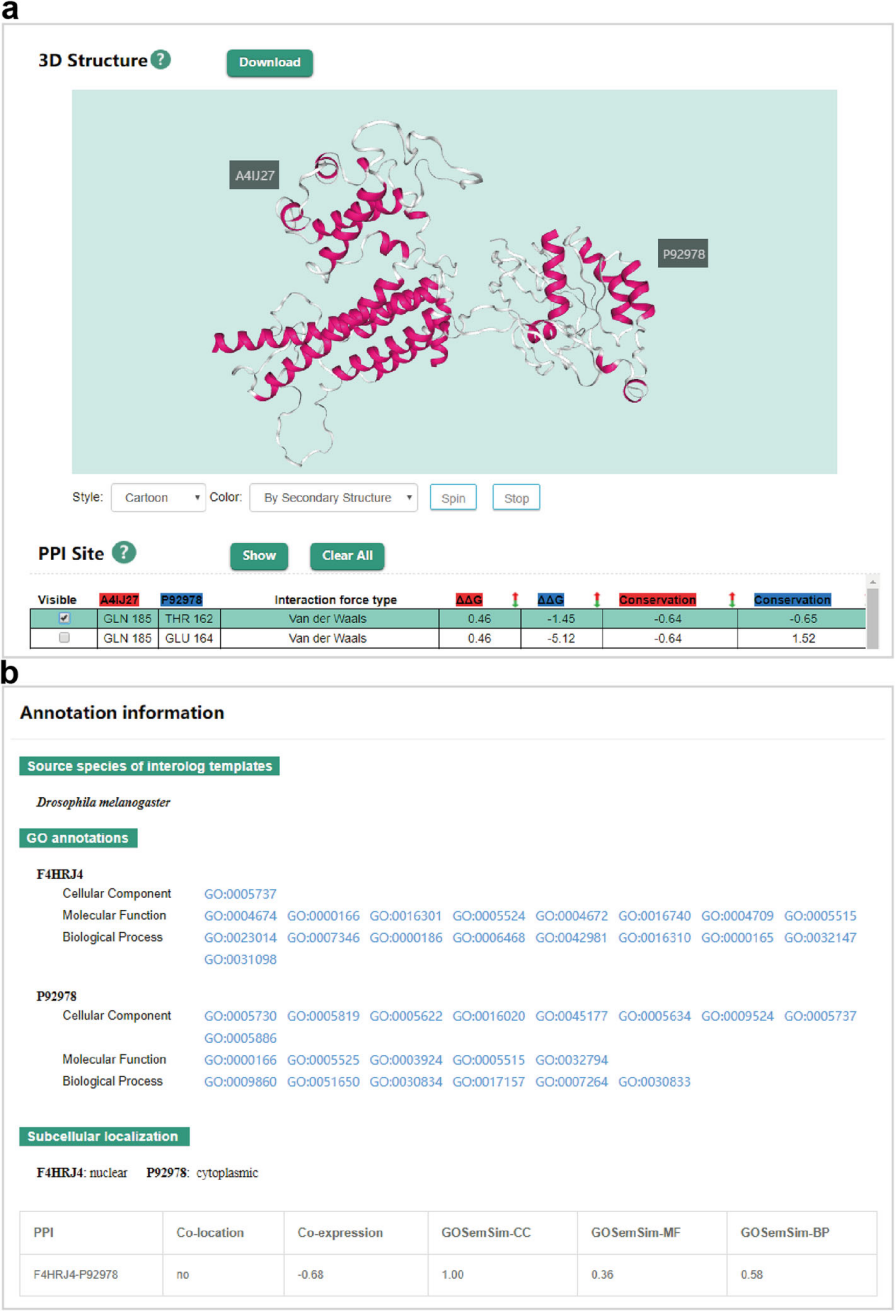
Complex structure and annotation information. **a** An example showing the predicted complex structure of an experimentally verified PPI. Users can select interested interaction sites in the table to display them on the complex structure as well as view the corresponding physicochemical properties listed in the table. **b** An example showing the annotation information for a predicted PPI. The source species of interolog templates, GO annotations and subcellular localizations are listed in the table. In addition, the corresponding similarities of GO annotations and gene expression profiles are also shown in the table

Compared to the previous version, PlaPPISite applies the new plug-in NGL to display protein complex structures, which has been widely used in many protein structure databases such as PDB. The utilization of the new plug-in adds a variety of colour schemes and molecular representations, such as backbone and spacefill. Complex structures can be rendered by any colour scheme and molecular representation and viewed from different angles through automatic rotation. In line with the previous version, the detailed interaction sites can be displayed on the complex structure, and the corresponding physicochemical properties are also listed, including bond type, conservation score and changes in Gibbs free energy (∆∆G) (Fig. 5a). Moreover, we provide the DDI/DMI annotations for the PPIs whose complex structures cannot be constructed. Notably, source species for interolog templates, GO annotations and subcellular localizations are listed for the predicted PPIs. Users may wish to use the calculated similarity measurements of GO annotations, gene expression profiles and subcellular localizations to further judge the reliability of PPIs (Fig. 5b).

### Visualization of PPI networks

Considering that the size of each plant interactome in PlaPPISite is large, we only provide subnetwork visualization for each PPI. In brief, for each PPI, PlaPPISite adds a subnetwork, which consists of the first-layer interactions involved in the two interacting proteins (Additional file 2: Figure S7). The subnetwork can be presented by various layouts and exported for further analysis. In the subnetwork, the edge length and node spacing can be adjusted as needed. Regarding the predicted PPIs in the subnetwork, note that the node is coloured by the subcellular localization and the edge is coloured by the gene expression similarity between two nodes.

### Prediction platform

To help users construct protein complex structures and assign interaction sites for their own PPIs that are not deposited in PlaPPISite, the online prediction platform has been built based on the HMPC method (Additional file 2: Figure S8a). As a result, the templates of two query proteins, the sequence identity and coverage between the template and query protein, the complex structure, and detailed interaction sites would be obtained (Additional file 2: Figure S8b).

### Reliability of predicted PPIs and predicted protein complex structures

To increase the coverage of these 13 plant interactomes, a large amount of predicted PPI data was collected in PlaPPISite, although the reliability of predicted PPIs is always controversial. Even though three pieces of indirect evidence and a direct comparison with a predicted *A. thaliana* interactome developed by [43] have been provided to prove the acceptable reliability of the PPI prediction, the predicted PPIs in PlaPPISite may inevitably contain large volumes of false positives. Two efforts have been made to effectively guide users to use the predicted PPI data properly. First, only the high-quality PPIs were retained and deposited in PlaPPISite. The high-quality here means the complex structures of those PPIs can be built up or DDIs/DMIs can be annotated. Second, the similarities of GO annotations, gene expression profiles and subcellular localizations for predicted PPIs are also presented to guide users to use the predicted PPIs properly.

Although bioinformatics algorithms for protein complex structure prediction have been widely developed, the reliability of predicted protein complex structures is also difficult to quantitatively assess. In our previous publication of AraPPISite [31], we mainly used 27 experimentally determined complex structures of *A. thaliana* PPIs as a test set to evaluate the performance of HMPC and PRISM. Although the size of the test set was very limited, the results showed that both HMPC and PRISM achieved a reasonable performance in constructing complex structures. Comparatively, the accuracy of HMPC outperformed that of PRISM.

**Fig. 6:**
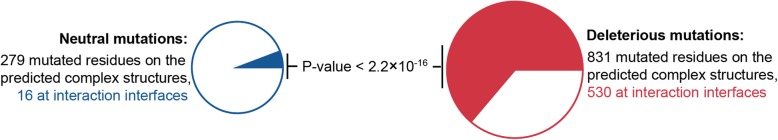
Deleterious mutations tend to occur significantly at the predicted interaction interfaces compared with neutral mutations (Fisher’s exact test, one-tailed *P*-value < 2.2 × 10^− 16^)

Moreover, we collected 4493 mutated sites of 995 *A. thaliana* proteins from two sources [51, 52] to further judge the quality of predicted interaction sites. The mutations were collected from manually collected mutations with phenotypic effects, which can be found in TAIR, and other mutations by using a literature search through Google Scholar. A total of 248 proteins containing 1110 mutated residues (279 neutral mutations and 831 deleterious mutations) were included in our predicted complex structures (Additional file 1: Table S6). As a result, 530 out of 831 deleterious mutations are located at the predicted interaction interface, whereas only 16 out of 279 neutral mutations occur at the interaction interface. Therefore, the deleterious mutations were significantly enriched at the predicted interaction interface compared with the neutral mutations (Fisher’s exact test, one-tailed *P*-value < 2.2 × 10^− 16^; Fig. 6). It has been well established that deleterious mutations are more likely located at the protein interaction interface compared with neutral mutations [53–55]. For instance, David and Sternberg (2015) reported the different distribution and properties of disease-causing single amino acid variations (SAVs) and polymorphisms within different structural regions [54]. They observed that 1960 out of 3283 human disease-causing SAVs are located at the interaction interface, whereas only 603 out of 1699 polymorphisms without known disease associations occur at the interaction interface. The results indicate that disease-causing SAVs are more likely to occur at the interaction interface compared with polymorphisms (Fisher’s exact test, one-tailed *P*-value < 2.2 × 10^− 16^), which is in line with our finding. Therefore, the above computational analysis added additional evidence to prove the reliability of the predicted interaction sites. Taken together, our current and previous computational analyses support the reasonable reliability of predicted complex structures and interaction sites.

## Conclusions

PlaPPISite is a freely available public resource that provides abundant PPI details for 13 plant species. At the structural level, PlaPPISite not only includes the 3D structures and interaction sites of experimental/predicted PPIs for 13 important plants but also lists the physicochemical properties and the residue conservation of interaction sites. Moreover, DDI/DMI information are also annotated for those PPIs whose 3D structures could not be successfully constructed. It should be emphasized that the PPI and interaction site information deposited in PlaPPISite may inevitably contain false positives, although we have conducted a series of computational experiments to intuitively provide evidence regarding the reliability of the predicted PPIs and protein complex structures. By taking the potential false positives in mind, we hope PlaPPISite can become an important data platform for accelerating our global understanding of plant interactomes. For instance, it can effectively guide experimental efforts such as mutagenesis to interrogate the functional roles of plant PPIs.

## Supplementary information

**Additional file 1: TableS1.** The PPI number distribution for the 13 plants in PlaPPISite. **Table S2.** The number of experimentally verified PPIs of the 13 plants. **Table S3.** The number of experimentally verified PPIs of six model organisms. **Table S4.** The GO annotation covrage for the 13 plants. **Table S5.** The subcellular co-localization proportion for the 13 plants. **Table S6.** The known mutated information associated with predicted interaction sites.

**Additional file 2: Figure S1.** The distribution of the average cellular component similarities for 1000 random networks and the predicted network. **Figure S2.** The distribution of the average biological process similarities for 1000 random networks and the predicted network. **Figure S3.** The distribution of the average molecular function similarities for 1000 random networks and the predicted network. **Figure S4.** The distribution of the average subcellular co-localization proportions for 1000 random networks and the predicted network. **Figure S5.** The distribution of the average expression profile similarities for 1000 random networks and the predicted network. **Figure S6.** Venn diagram showing the numbers of overlapping PPIs among two predicted PPI sets and one experimental PPI set. **Figure S7.** The primary subnetwork of PPI. Users can export the subnetwork alternatively for further analysis. **Figure S8.** A prediction platform for complex structure construction and interaction site assignment. (a) The prediction platform interface. Users can submit two protein sequences of a query PPI to retrieve the complex structure and the corresponding interaction sites. (b) A prediction result example. The predicted complex structure and the corresponding interaction sites can be downloaded on this page.

## Data Availability

The database is freely available via http://zzdlab.com/plappisite/index.php.

## Abbreviations

∆∆G: Changes in Gibbs Free Energy
3did: the Database of 3D Interacting Domains
DDIs: Domain-Domain Interactions
DMIs: Domain-Motif Interactions
GEO: Gene Expression Omnibus
GO: Gene Ontology
HMPC: Homology Modelling of Protein Complex
PDB: Protein Data Bank
PPIs: Protein-Protein Interactions
PRISM: Protein Interactions by Structural Matching
