# Supplementary material for: PlaPPISite: a comprehensive resource for plant protein-protein interaction sites
Source: BMC Plant Biol. 2020 Feb 6;20:61. doi: 10.1186/s12870-020-2254-4 (PMC7006421; doi:10.1186/s12870-020-2254-4)
Supplement: Supplementary file 2 — Additional file 2: Figure S1. The distribution of the average cellular component similarities for 1000 random networks and the predicted network. Figure S2. The distribution of the average biological process similarities for 1000 random networks and the predicted network. Figure S3. The distribution of the average molecular function similarities for 1000 random networks and the predicted network. Figure S4. The distribution of the average subcellular co-localization proportions for 1000 random networks and the predicted network. Figure S5. The distribution of the average expression profile similarities for 1000 random networks and the predicted network. Figure S6. Venn diagram showing the numbers of overlapping PPIs among two predicted PPI sets and one experimental PPI set. Figure S7. The primary subnetwork of PPI. Users can export the subnetwork alternatively for further analysis. Figure S8. A prediction platform for complex structure construction and interaction site assignment. (a) The prediction platform interface. Users can submit two protein sequences of a query PPI to retrieve the complex structure and the corresponding interaction sites. (b) A prediction result example. The predicted complex structure and the corresponding interaction sites can be downloaded on this page. [file 12870_2020_2254_MOESM2_ESM.docx]

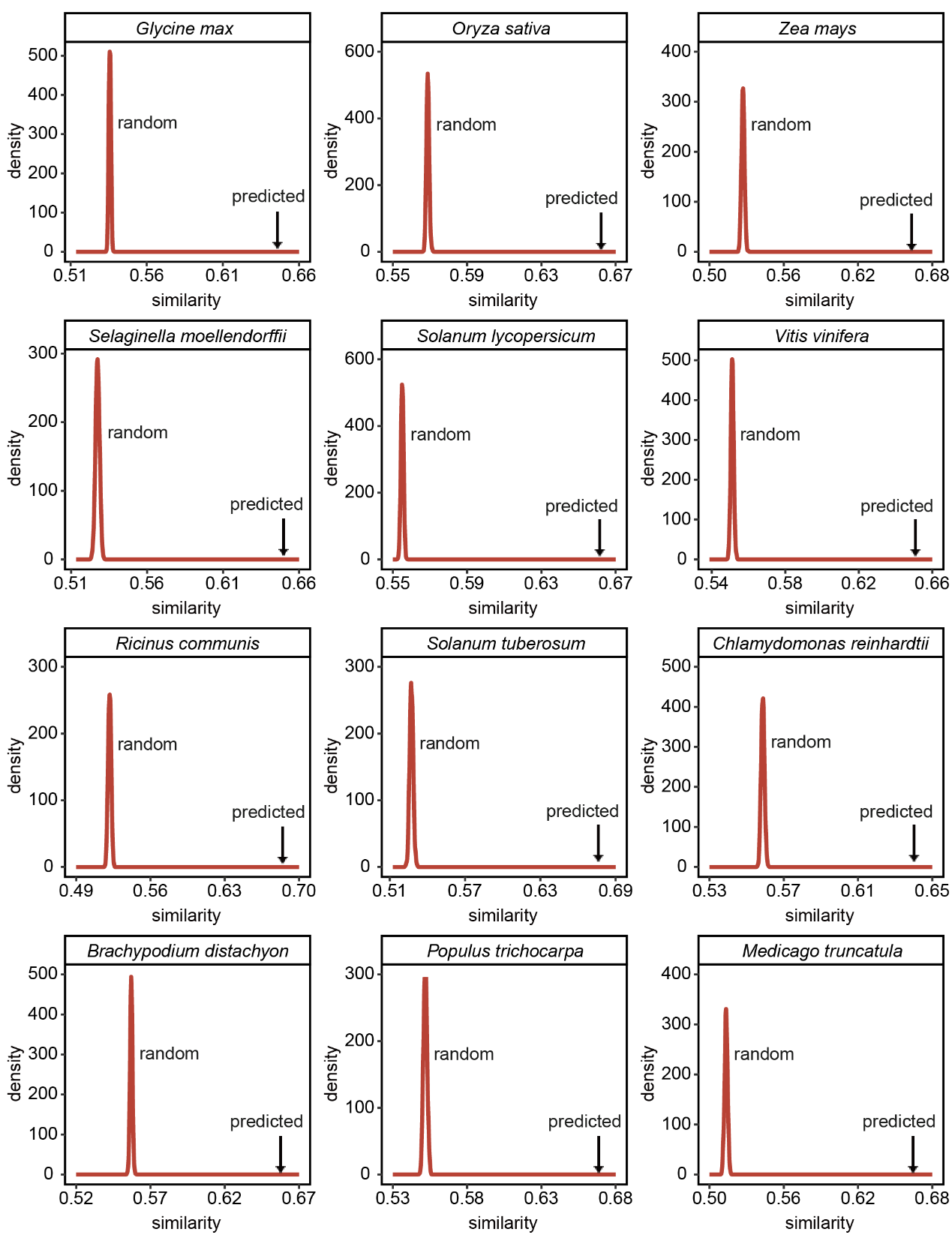


Figure S1 The distribution of the average cellular component similarities for 1,000 random networks and the predicted network.


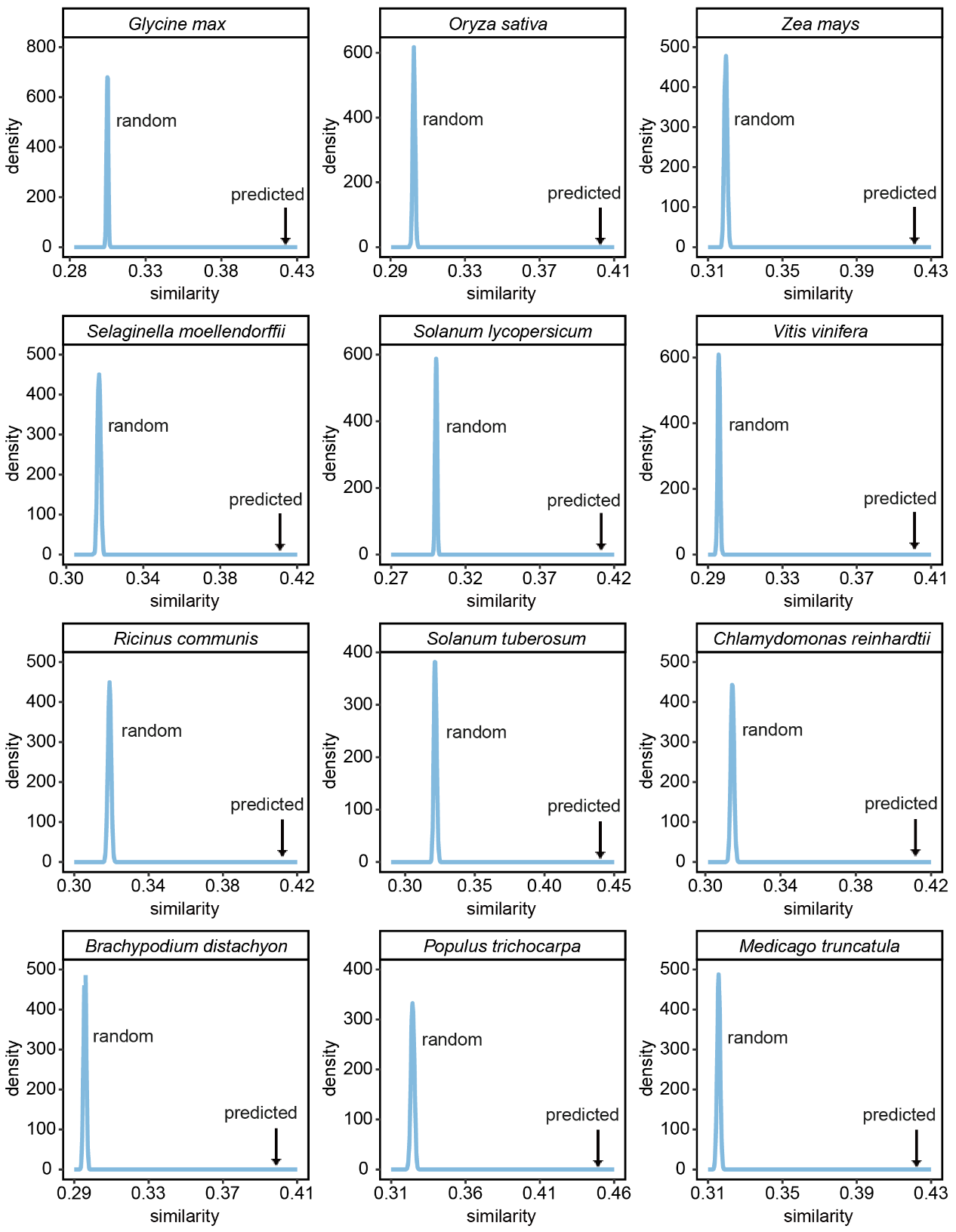


Figure S2 The distribution of the average biological process similarities for 1,000 random networks and the predicted network.


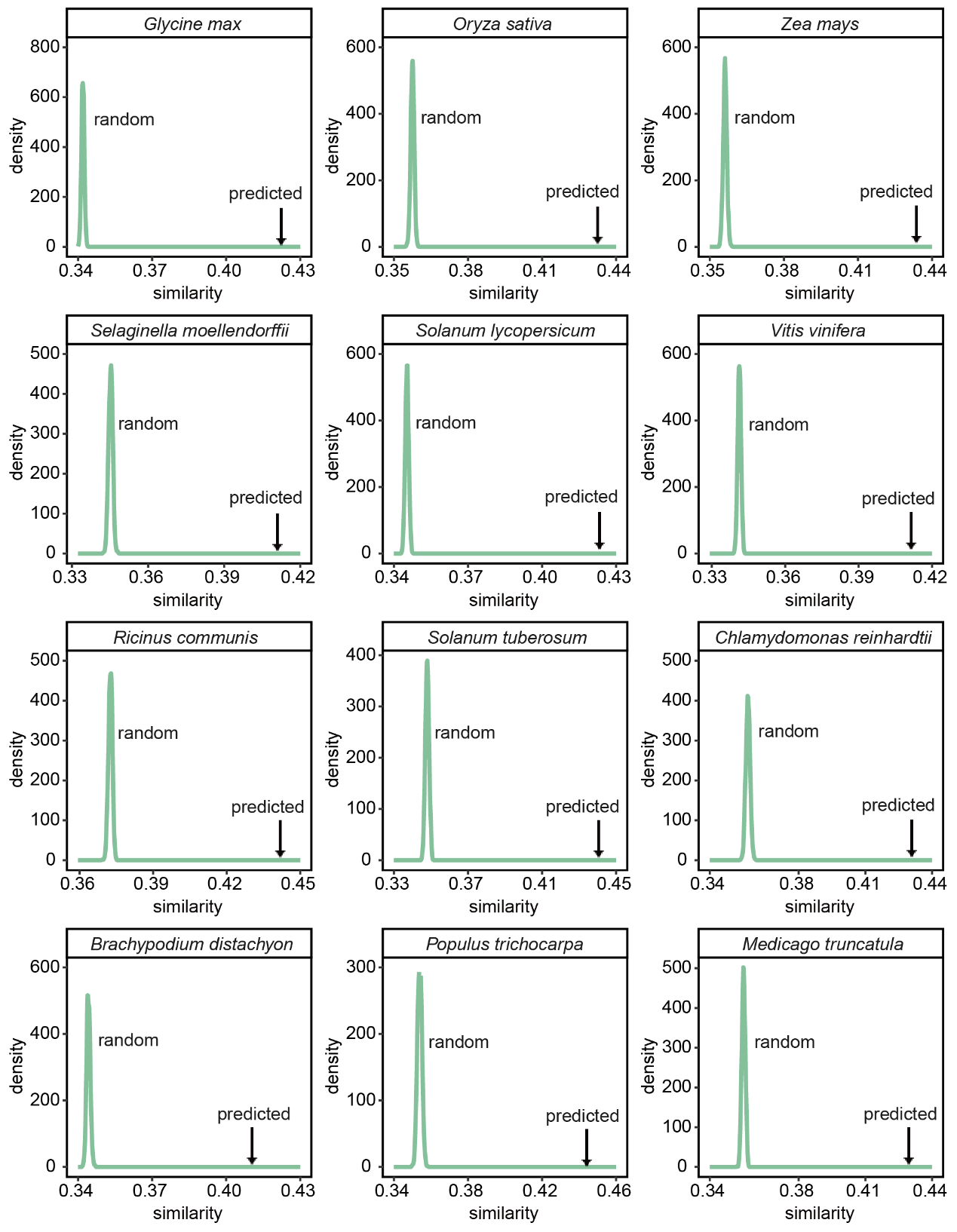


Figure S3 The distribution of the average molecular function similarities for 1,000 random networks and the predicted network.


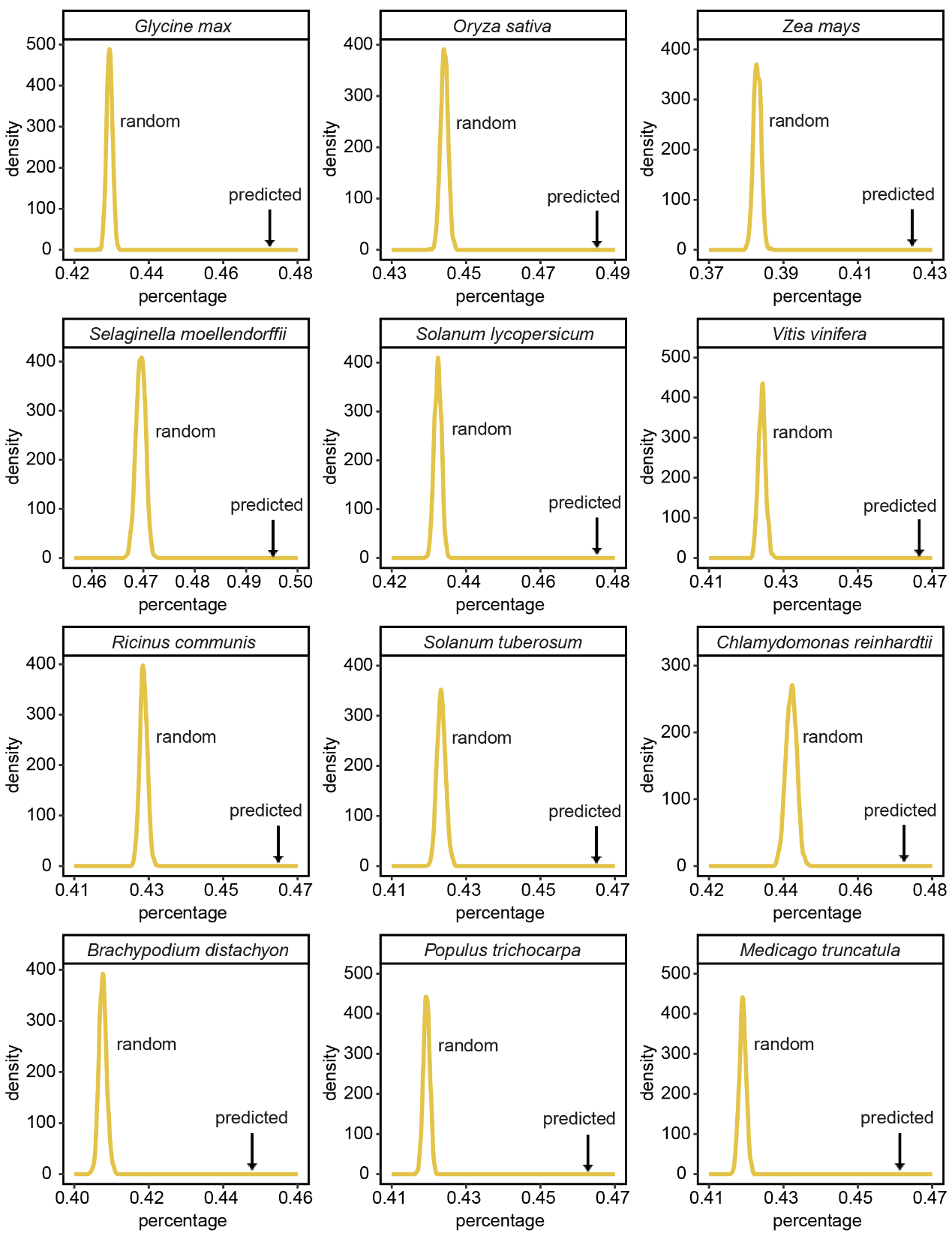


Figure S4 The distribution of the average subcellular co-localization proportions for 1,000 random networks and the predicted network.


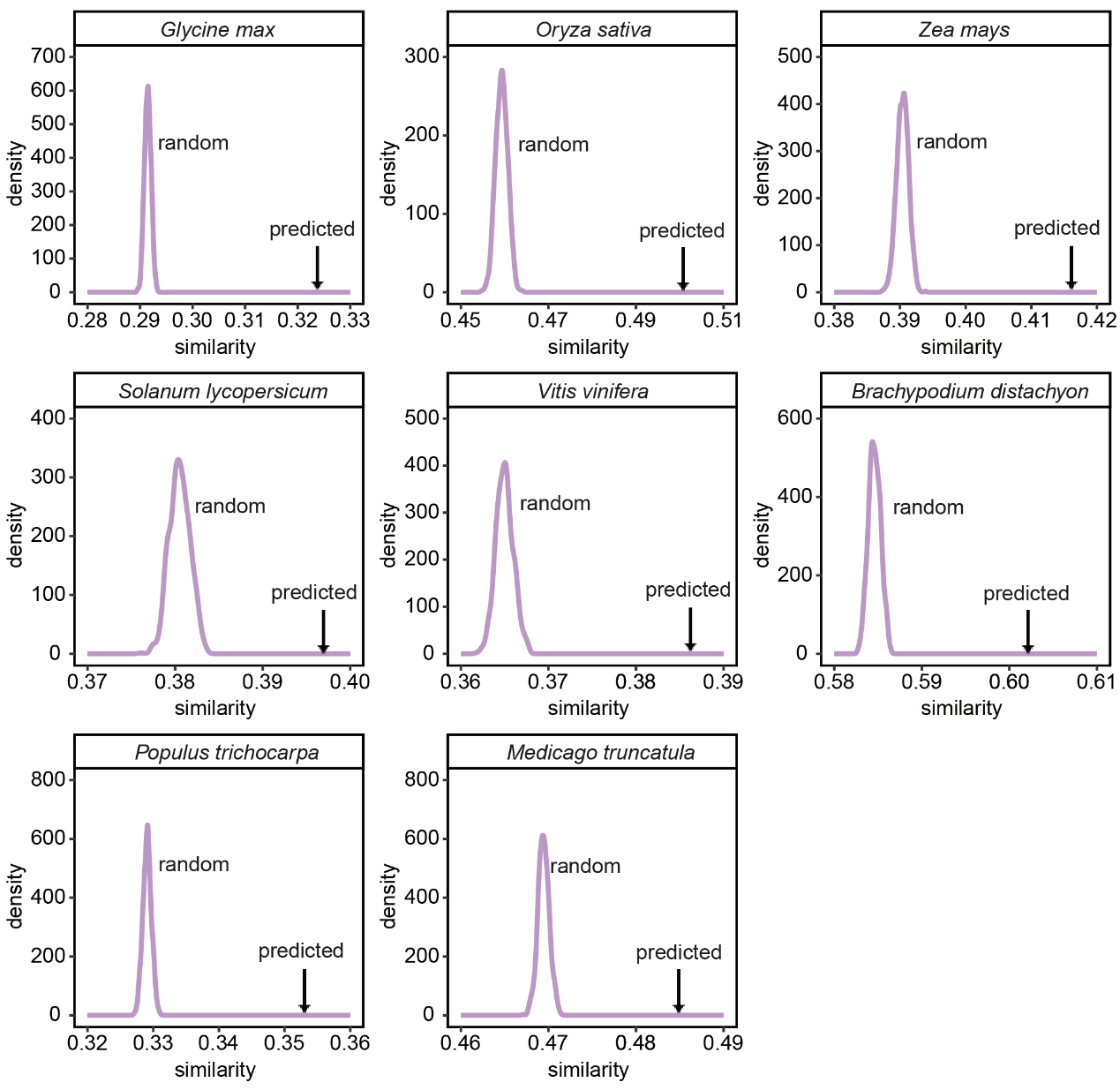


Figure S5 The distribution of the average expression profile similarities for 1,000 random networks and the predicted network.

***
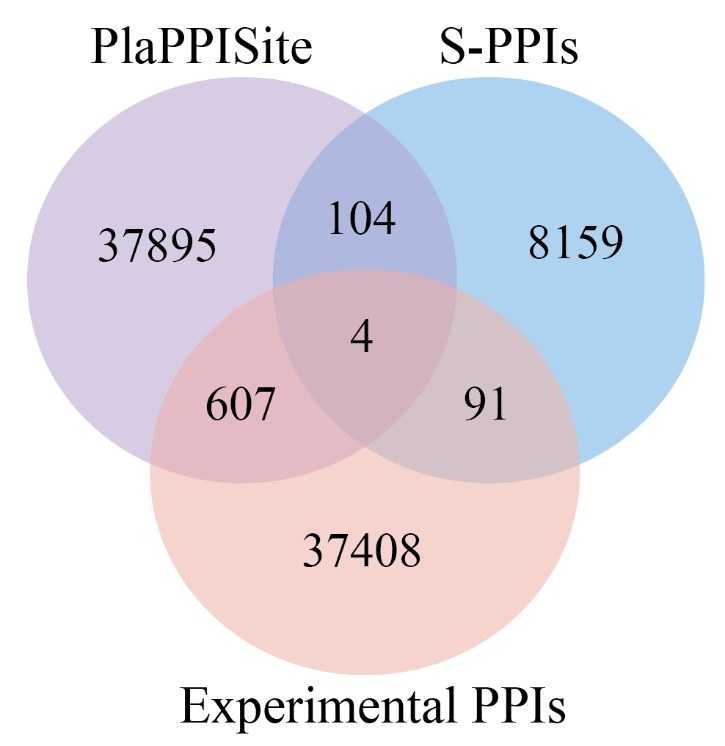
***

Figure S6 Venn diagram of overlapping PPIs among two predicted PPI sets and one experimental PPI set.


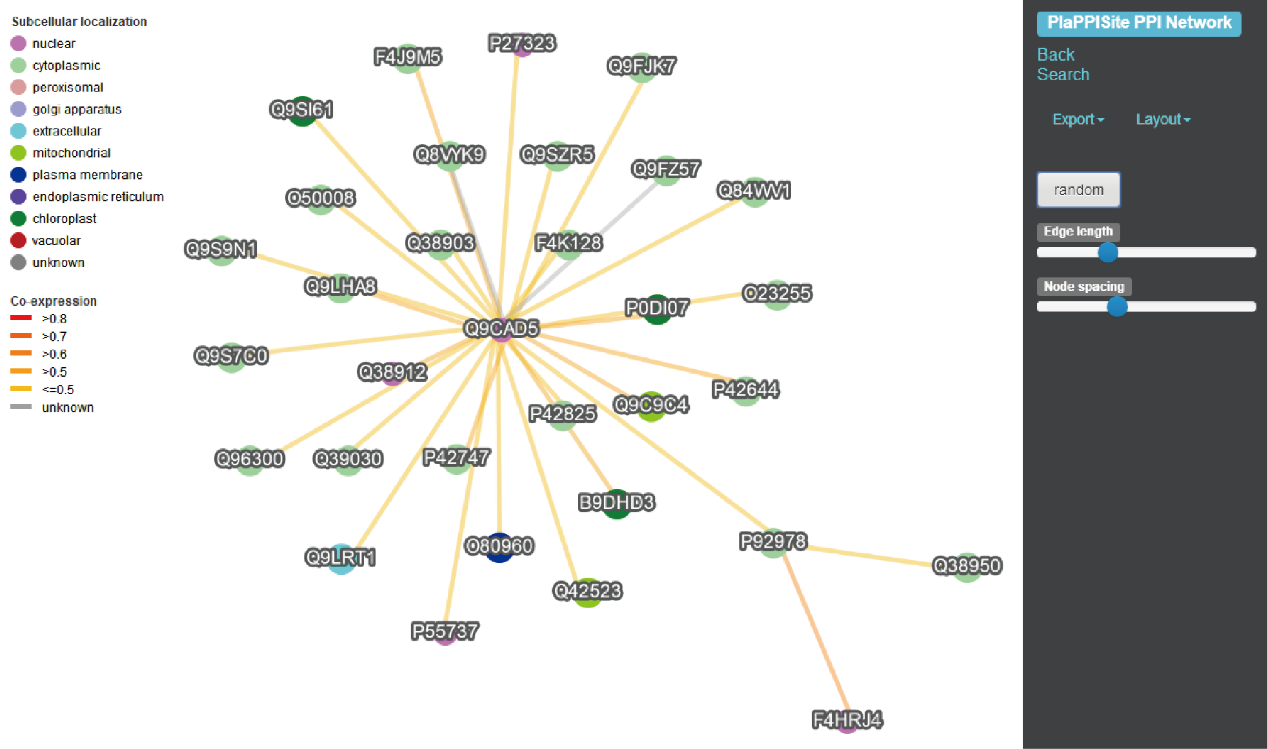


Figure S7 The primary subnetwork of PPI. Users can export the subnetwork alternatively for further analysis.


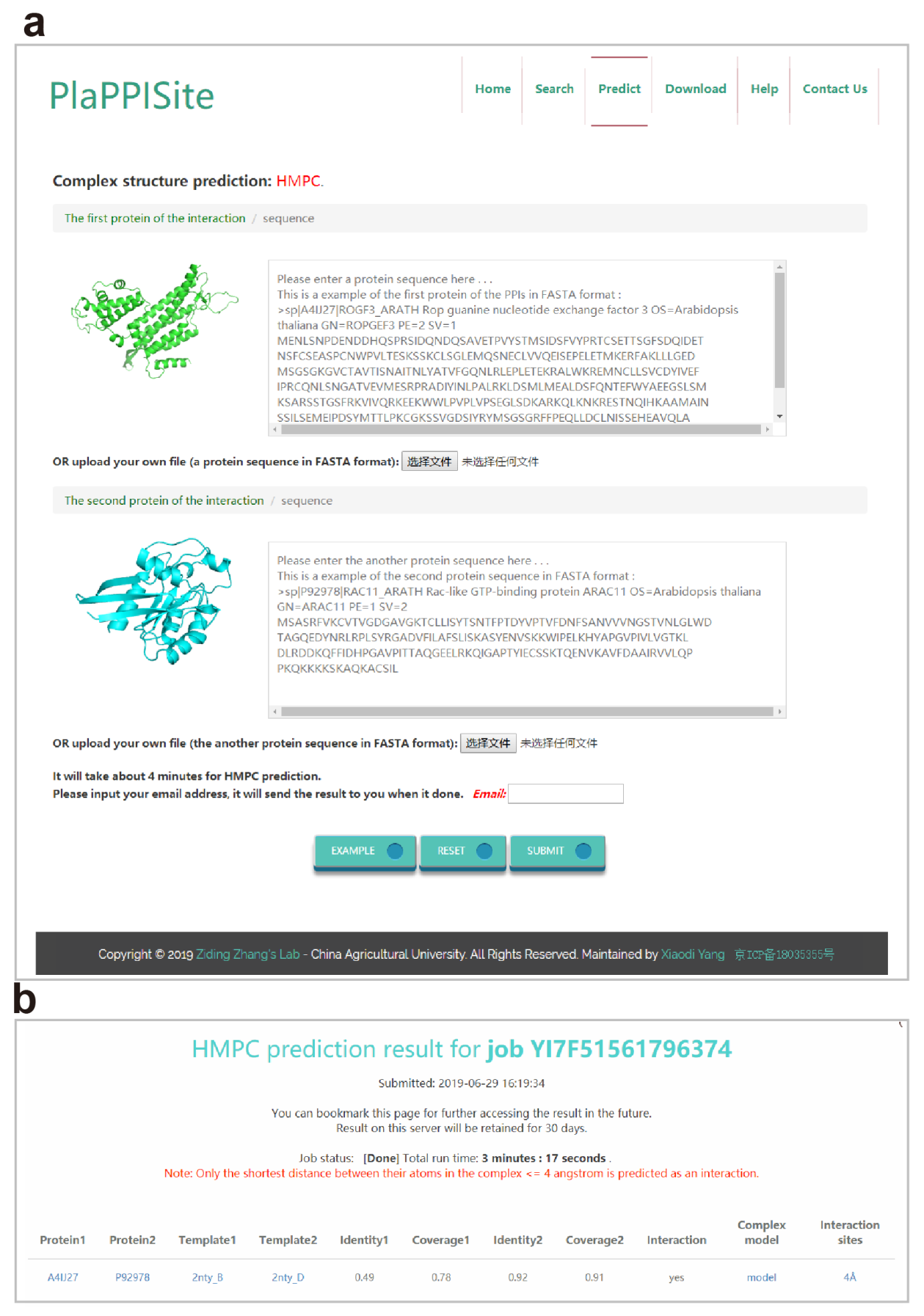


Figure S8 A prediction platform of complex structures and interaction sites.
